# An Optimized In-House Protocol for *Cryptococcus neoformans* DNA Extraction from Whole Blood: “Comparison of Lysis Buffer and Ox-Bile Methods”

**DOI:** 10.3390/jof11060430

**Published:** 2025-06-04

**Authors:** Fredrickson B Wasswa, Kennedy Kassaza, Kirsten Nielsen, Joel Bazira

**Affiliations:** 1 Department of Microbiology and Parasitology, Mbarara University of Science and Technology, Mbarara P.O. Box 1410, Uganda; fredricksonwasswa@gmail.com (F.B.W.); kkassaza@must.ac.ug (K.K.); 2 Department of Microbiology & Immunology, University of Minnesota, Minneapolis, MN 55455, USA; knielsen@umn.edu

**Keywords:** ox-bile, lysis buffer, *Cryptococcus neoformans* in blood

## Abstract

*Cryptococcus neoformans (C. neoformans)* is a capsulated yeast that enters the body through inhalation and migrates via the bloodstream to the central nervous system, causing cryptococcal meningitis. Diagnosis methods are culture, serology, and India ink staining, which require cerebrospinal fluid (CSF) or whole blood. Molecular methods are used for epidemiological studies and require expensive commercial DNA extraction kits. This study aimed to develop an economical in-house method for extracting *C. neoformans* DNA from whole blood. *C. neoformans* cells of varying McFarland standards were spiked into expired blood, then lysed using laboratory-prepared lysis buffer and ox-bile solution, followed by organic DNA extraction. Ordinary PCR targeting the CNAG 04922 gene was performed. To determine the limit of detection, serial dilutions of *C. neoformans* were made, and DNA extraction was performed on other parts cultured on yeast extract peptone dextrose agar to determine colony-forming units (CFU). The lysis buffer method successfully extracted DNA from as low as the average of 62 CFU in 0.9 mL of expired blood with superior quality and high yield compared to ox-bile. The lysis buffer method yielded higher DNA quality and quantity than ox-bile and detected low concentrations of *C. neoformans* in expired blood. This method presents a cost-effective alternative for molecular diagnosis in resource-limited settings.

## 1. Introduction

*Cryptococcus neoformans* is an encapsulated budding yeast [1,2,3,4] that causes opportunistic infections in immunosuppressed individuals [5], particularly those with HIV/AIDS [6,7], organ transplants, or undergoing chemotherapy [8]. Inhaled fungal spores establish an initial infection in the lungs [1,9] before disseminating via the bloodstream to the central nervous system (CNS), leading to cryptococcal meningitis [10,11,12,13,14,15].

Traditionally, *C. neoformans* is diagnosed through culture and India ink staining of CSF [16,17,18]. While accurate, these methods are invasive and have low sensitivity [19,20]. More recently, the cryptococcal antigen (CrAg) lateral flow assay (LFA) has revolutionized diagnosis [21,22,23,24] by detecting bloodstream infections before clinical presentation [25]. However, CrAg LFA does not allow downstream microbial analysis like molecular techniques, making culture the gold standard despite its time constraints [23,26,27,28,29,30,31].

Molecular techniques such as PCR provide high sensitivity for rapid diagnosis [32,33,34,35], but their reliance on commercial DNA extraction kits limits affordability in low-resource settings [36,37]. This study aims to develop a cost-effective, in-house molecular diagnostic assay for *C. neoformans* DNA extraction from whole blood, complementing existing CrAg LFA screening.

## 2. Materials and Methods

Study site: The study was conducted at the Genomic Translation and Research Laboratory (GTRL) and the Microbiology Laboratory at the Microbiology Department of Mbarara University of Science and Technology (MUST), Uganda

Strain and Culture: The H99 strain of *C. neoformans* (University of Minnesota, Minneapolis, MN, USA) was cultured on yeast extract peptone dextrose (YPD) agar (Research products international, RPI, Mt. Prospect, IL, USA) and incubated at 37 °C for 48 h.

Blood Samples and spiking: Expired blood obtained from the Mbarara Regional Referral Hospital blood bank was stored at 4–8 °C. This blood is not suitable for human transfusion but is kept at above-temperature before being burnt. This condition still keeps the cell components intact, which could still be used to mimic blood cell constituents, although there could be variations with normal blood cells in humans that have not been stored [38]. Suspensions of *C. neoformans* 0.5, 1, 2, 3, 4, and 5 McFarland were prepared using DENSIMAT (Biomerieux Italia S.P.A, Bagno a Ripoli (FI) Italy) and spiked into 0.9 mL and 3.9 mL aliquots of expired blood. The above McFarlands correspond to 1.99 × 10^6^, 4.05 × 10^6^, 7.72 × 10^7^, 2.5 × 10^8^, 1.02 × 10^9^, and 1.2 × 10^10^ cells/mL, respectively. The above *C. neoformans* cell counts were performed using a Superior Marienfeld count chamber (Fisher Scientific, Schwerte Germany) using the formula in [39].

Workflow for *Cryptococcus neoformans* DNA extraction from whole blood using Ox bile and In-House Lysis Buffer method.

**Figure 1:**
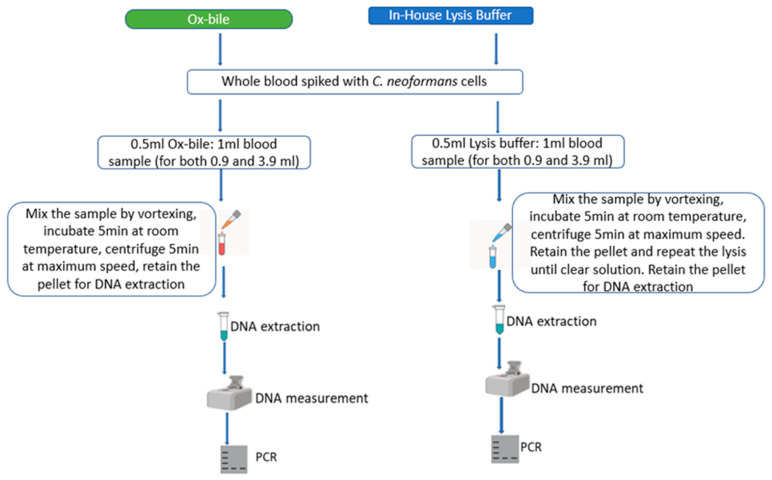
Workflow for *Cryptococcus neoformans* DNA extraction from samples lysed either by ox-bile or in-house lysis buffer. The processes included RBC lysis by the above methods, DNA extraction, quality measurement, PCR, and gel electrophoresis. Created with Microsoft PowerPoint [40].

Red Blood Cell Lysis: Two methods were evaluated, as shown in Figure 1.

1 Lysis Buffer: Prepared in-house (5 mL of 2 M Tris pH 7.6, 5 mL of 1 M MgCl_2_, 3.3 mL of 3 M NaCl, and up to 1000 mL of distilled water).
2 Ox-Bile Solution: 10 g ox-bile powder (made from cow by Solarbio Beijing China), known for lysing red blood cells [41] was dissolved in 100 mL of distilled water.

For the lysis buffer, after adding 500 µL of lysing buffer to 1 mL of samples (RBCs mixed with *Cryptococcus neoformans* cells). The samples were vortexed for 1 min to form a uniform mixture, incubated for 5 min at room temperature, centrifuged at a maximum speed of 21,130 rcf for 5 min, and the supernatant was poured off. To the sediment, lysis buffer was added, and the processes were repeated as above until RBCs were completely lysed. However, with subsequent lysing, the volume of lysis buffer can be increased to up to 1.5 mL in the tube. The lysed sediment was reduced to ≅150 µL carefully using a 1000 µL pipette tip and was ready for *C. neoformans* DNA extraction.

For ox-bile, after adding 500 µL of the ox-bile to 1 mL of the samples, the samples were vortexed for 1 min to form a uniform mixture, incubated for 5 min at room temperature, centrifuged at maximum speed of 21,130 rcf for 5 min, the supernatant was poured off, and the lysed sediment was reduced to ≅ 150 µL carefully using 1000 µL pipette tips, ready for *C. neoformans* DNA extraction.

Too much volume of *C. neoformans* sediment would affect the concentration of DNA extraction buffer, yet the *C. neoformans* are sedimented at the bottom of a 1.5 mL tube.

DNA Extraction: Suspend in 500 µL of extraction buffer (0.1 M Tris pH8.0, 50 mM EDTA, 1% SDS) with 3 mm of Solid-glass beads Lot#3110 (Sigma-Aldrich, Taufkirchen, Germany) into the lysed sediment of ≅150 µL comprising *Cryptococcus neoformans*. Vortex for 1 min and allow the tube to rest for 1 min at room temperature to allow beads to settle. Add 275 µL of 7 M Ammonium Acetate pH 7.0 and incubate 30 min at 65 °C (dry bath). Incubate 5 min on ice and then add 500 µL of chloroform, vortex for 1 min, followed by centrifugation at maximum speed for 5 min. Transfer aqueous layer of DNA (topmost, ensuring no debris is pipetted using 200 µL pipette tips) to a 1.5 mL new labeled tube and dispose of the old tube. Add 1 mL of Isopropanol and incubate 5 min at room temperature (with mixing by inverting the tube several times) and centrifuge at a maximum speed of 21,130 rcf for 7 min. Remove Isopropanol and wash the pellet with 1000 µL of 70% Ethanol, then centrifuge at maximum speed for 3 min. Remove Ethanol by decanting, followed by 1 min centrifugation, then remove the remaining Ethanol with a pipette tip. Allow DNA to air dry, then add 50 µL of TE buffer for resuspension of DNA. Store extracted DNA at −20 °C.

Quality and Quantification: DNA quality and concentration were assessed using a NanoDrop spectrophotometer (NanoDrop lite spectrophotometer, Thermo Scientific-168 Third Avenue, Waltham, MA, USA 02451). Good quality DNA lies between 1.8 and 2.0.

Primer design: Primers were designed using Snapgene software targeting the CNAG 04922 gene located on chromosome 10 of the *Cryptococcus neoformans* genome, which is responsible for a cysteine protease that is thought to have an impact on the patient out. Primer sequences were as follows: forward primer 5′-CATGGATCGAGGGGAACGA-3′ and reverse primer 5′-TCATCAGTTGGATGGATCATGCT-3′, respectively, manufactured by Inqaba Biotec Limited, Pretoria South Africa.

Selectivity of the primer: *C. neoformans* was run along with other species of fungi; *Candida albicans* ATCC 10234, *Candida* sp., *Aspergillus* sp., and *Trichophyton mentagrophytes,* identified phenotypically, were obtained from the Mycology Laboratory at the Department of Microbiology, MUST. *Staphylococcus aureus* ATCC 25923 and *Escherichia coli* ATCC 25922 were obtained from the Microbiology Laboratory at the Department of Microbiology. The same processes of DNA extraction, quality, and quantification, and PCR conditions were applied. This was performed to determine whether primers were selective to the *C. neoformans* genome alone.

Limit of Detection (LOD) and PCR: Serial dilutions were also cultured on YPD to determine CFU per each serial dilution, which would be used in comparison with PCR amplification results of the same serial dilutions, 0.5 McFarland (0.5 ≅ 1–5 × 10^6^ cells/mL) [42] of 1:10 of *Cryptococcus neoformans*. A volume of 100 µL of these serial dilutions was cultured on the PYD media in duplicates on the same plate after dividing it into two equal portions. The plates were cultured using conditions described above. After 48 h, colonies were counted from both sides, and the average was taken. The PCR targeted the CNAG 04922 gene to establish the lowest detectable concentration via PCR. The master mix was made of 1× standard PCR buffer (10 mM Tris-HCl, 50 mM KCl, 1.5 mM MgCl_2_, and pH 8.3 at 25 °C), 0.2 mM dNPTs 1.25 Units Taq polymerase (BioLabs New England, Ipswich, MA, USA ) 0.2 uM of forward primer, 0.2 uM of reverse primer, 15.75 µL nuclease free water, and 5 µL of template DNA in a total volume of 25 µL. PCR was performed under the following conditions on the extracted *cryptococcus neoformans* DNA 04922 gene in *Cryptococcus neoformans*: 95 °C for 3 min, 95 °C for 30 s, 56 °C for 30 s, 68 °C for 1 min for 40 cycles, and the final extension at 68 °C for 5 min, amplifying the CNAG 04922 gene using MULTIGene OPTIMAX thermocycler (Labnet International, Inc. Edison, NJ, USA). After PCR, band detection was performed using 1.5% agarose for gel electrophoresis at 200 V for 45 min.

## 3. Results and Discussion

Comparison of RBC Lysis Methods: The in-house lysis buffer consistently yielded a higher DNA purity (A260/A280 of 1.87–1.90) and concentration (up to 853.47 ng/µL in 3.9 mL blood) compared to ox bile (A260/A280 of 1.46–1.85, DNA yield of up to 266.73 ng/µL).

The mean of the duplicate sample reading was calculated, as it gives the central tendency of the results. A paired samples *t*-test showed that DNA yield increases significantly with an increase in blood volume from 0.9 mL to 3.9 mL, when using the lysis buffer extraction method (*p* = 0.003).

DNA Quality and Quantity Measurements: Lysis buffer yielded a high quantity of DNA, while ox bile yielded a relatively low quantity of DNA. For high volumes, still, ox bile had a reduced yield of DNA. In terms of quality, using a nanodrop spectrophotometer at (A260/A280), the lysis buffer had good quality DNA 1.87–1.90, while ox bile had a poor quality of DNA below 1.80, i.e., 1.46–1.85, as shown in Table 1.

**Figure 2:**
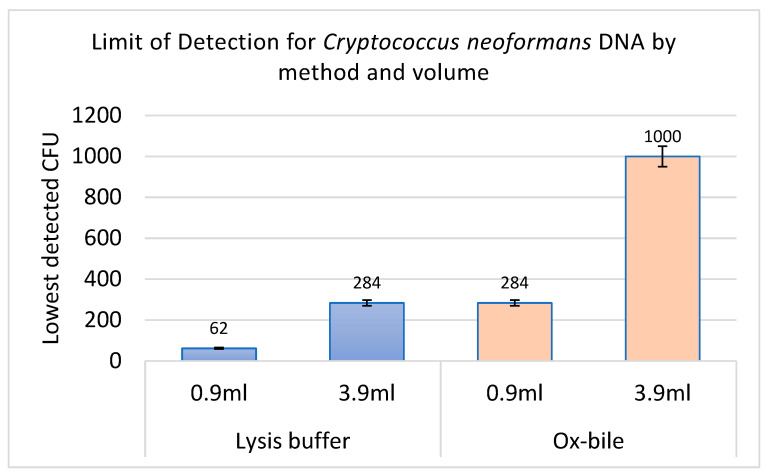
Error bar of limit of detection (CFU/mL) for *C. neoformans* from culture, which corresponded to the DNA extracted from spiked whole blood with *C. neoformans.* The average of duplicate serial dilutions culture growth is presented; for the fourth serial dilution (1 × 10^4^), 62, third serial dilution (1 × 10^3^), 284, second serial dilution (1 × 10^2^), 1000 CFU, respectively. The in-house lysis buffer method detected as low as the average of 62 CFU in 0.9 mL, while the ox-bile method detected an average of 284 CFU. Average values were used because they represent the central tendency of the results.

**Figure 3:**
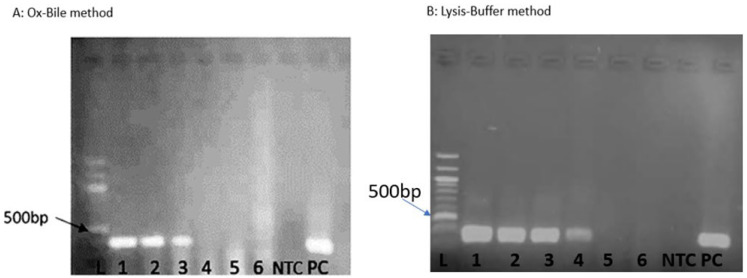
PCR detection of *Cryptococcus neoformans* DNA in serial dilutions of whole blood samples. (**A**): Ox-bile method showing bands up to the 3rd dilution. (**B**): Lysis buffer method showing bands up to the 4th dilution. Lane L = Ladder, 1–6 = Serial dilutions i.e., 1 = 1 × 10^1,^ 2 = 1 × 10^2^, 3 = 1 × 10^3^, 4 = 1 × 10^4^, 5 = 1 × 10^5^, and 6 = 1 × 10^6^, NTC = No template control, PC = Positive control.

Limit of Detection: Lysis buffer successfully extracted detectable DNA from as low as the average of 62 CFU/mL, whereas ox-bile had a detection limit of the average of 284 CFU/mL, as shown in Figure 2, which corresponds to the fourth dilution and third dilution, respectively, (Figure 3) by PCR detection. The increase in the blood volume was to demonstrate the effect of much blood on a limited number of *C. neoformans* cells, implying that in cases of suspected few *C. neoformans* cells, blood volume can be increased to increase the chances of detection. The superior performance of the lysis buffer is attributed to its multiple washing steps, reducing PCR inhibitors, like in [43].

Selectivity of the primer: Only *C. neoformans* was amplified, while the candida, molds, and both Gram-positive and negative bacteria did not amplify showing the primers are selective to the *C. neoformans* genome only. Figure S1 attached to the Supplementary File.

Culture-Based Growth Analysis: Culturing confirmed *C. neoformans* presence up to the sixth serial dilution (1 × 10^6^ CFU/mL). PCR detection was not possible at this dilution, highlighting the culture’s higher sensitivity at extremely low fungal burdens.

Clinical and Diagnostic Implications: This method offers a cost-effective alternative to commercial DNA extraction kits, making molecular diagnosis more accessible in low-resource settings [36,37]. The improved DNA purity enhances downstream applications such as sequencing and antimicrobial resistance profiling [44]. The assay can be used in a limited resource setting due to the challenge of extra cost for probes associated with real-time PCR [45].

Study limitation: This study needed to be applied to clinical blood samples for its validation; however, it was not possible to have such samples during the study period because they were not among the archived samples. Therefore, further validation with clinical samples is suggested.

## 4. Conclusions

The in-house lysis buffer method provides a cost-effective, high-yield alternative for *C. neoformans* DNA extraction from whole blood. It outperforms ox-bile in both DNA quality and sensitivity, making it a suitable option for molecular diagnostics in resource-limited settings.

## 5. Recommendation

Further studies should evaluate this method in clinical samples and assess its performance in hemolyzed blood specimens.

## Figures and Tables

**Table 1:** Summarizing the DNA purity and quantity extracted using the lysis buffer and ox bile of 0.9 mL and 3.9 mL volume of blood from the varying concentrations of *C. neoformans*.

			DNA Quality (A260/A280)			DNA Quantity (ng/µL)		
Method	*C. neoformans* Cells	Blood Volume (mL)	Mean (95% CI)	SD (95% CI)	*p*-Value	Mean (95% CI)	SD (95% CI)	*p*-Value
Lysis buffer	1 × 10^1^–10 × 10^4^	0.9	1.88 (1.87–1.89)	0.014 (0.008–0.018)	0.341	290.17 (267.17–310.08)	32.188 (17.07–38.13)	0.003
	1–5 McFarland	3.9	1.873 (1.87–1.88)	0.013 (0–0.018)		853.47 (624.86–999.7)	285.95 (38.6–406.5)	
Ox-bile	1 × 10^1^–10 × 10^4^	0.9	2.36 (1.69–3.57)	1.676 (0.015–2.46)	0.366	199.28 (110.79–268.19)	122.19 (19.99–143.07)	0.656
	1–5 McFarland	3.9	1.84 (1.833–1.85)	0.01 (0.004–0.015)		266.73 (173.85–359.09)	137.75 (64.132–179.49)	

## Data Availability

The original data presented in the study are openly available in, DOI: 10.6084/m9.figshare.29212502.

## Supplementary Materials

The following supporting information can be downloaded at https://www.mdpi.com/article/10.3390/jof11060430/s1, Figure S1: PCR detection of *C. neoformans* alongside with other organisms to determine the selectivity of the primers Lane 1—Ladder, 2—*C. neoformans*, 3—*Candida albicans* ATCC 10234, 4—*Candida* sp., 5—*Aspergillus* sp., 6—*Trichophyton mentagrophytes*, 7—*Staphylococcus aureus* ATCC 25923, 8—*Escherichia coli* ATCC 25922, 9—NTC—No template control, PC—Positive control. The primers were only selective to *C. neoformans* DNA.

## Abbreviations

The following abbreviations are used in this manuscript:

CSF	Cerebrospinal fluid
CFU	Colony-forming units
CNS	Central nervous system
CrAg	Cryptococcal antigen
LFA	Lateral flow assay
LOD	Limit of Detection
GTRL	Genomic Translation and Research Laboratory
MUST	Mbarara University of Science and Technology
*C. neoformans*	*Cryptococcus neoformans*
YPD	Yeast extract peptone dextrose
