# Supplementary material for: An Optimized In-House Protocol for Cryptococcus neoformans DNA Extraction from Whole Blood: “Comparison of Lysis Buffer and Ox-Bile Methods”
Source: J Fungi (Basel). 2025 Jun 4;11(6):430. doi: 10.3390/jof11060430 (PMC12194009; doi:10.3390/jof11060430)
Supplement: Supplementary file 1 [file jof-11-00430-s001.zip › jof-3611104-supplementary.pdf]

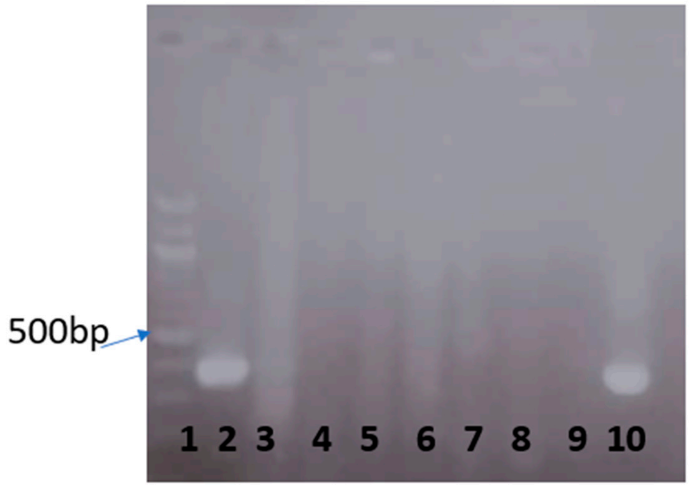

Figure S1: PCR detection of *C. neoformans* alongside with other organisms to determine the selectivity of the primers Lane 1 - Ladder, 2-*C. neoformans* 3- *Candida albicans* ATCC 10234, 4- *Candida sp*, 5- *Aspergillus sp*, 6- *Trichophyton mentagrophytes* 7- *Staphylococcus aureus* ATCC 25923, 8-*Escherichia coli* ATCC 25922, 9- NTC- No template control, PC- Positive control. The primers were only selective to *C. neoformans* DNA
